# Household Health and Cropland Abandonment in Rural China: Theoretical Mechanism and Empirical Evidence

**DOI:** 10.3390/ijerph16193588

**Published:** 2019-09-25

**Authors:** Xin Deng, Miao Zeng, Dingde Xu, Feng Wei, Yanbin Qi

**Affiliations:** 1 College of Economics, Sichuan Agricultural University, #211, Huimin Road, Chengdu 611130, China; dengxin@sicau.edu.cn (X.D.); weifeng@sicau.edu.cn (F.W.); 2 School of Economics, Sichuan University, #24, Yihuannan Road, Chengdu 610065, China; zengmiao@stu.scu.edu.cn; 3 Sichuan Center for Rural Development Research, College of Management, Sichuan Agricultural University; #211, Huimin Road, Chengdu 611130, China; dingdexu@sicau.edu.cn

**Keywords:** household health, cropland abandonment, rural medical services, China

## Abstract

Prior studies have fully explored the impacts of rural labor migration on land use forms. In contrast to prior studies, this study focuses on the health status of rural households and its quantitative impacts on cropland abandonment (CA). More specifically, under the guidance of the theoretical mechanism of “household health affects CA by labor supply”, this study employs survey data from 8031 households collected in 27 Chinese provinces in 2014 to explore the quantitative impacts of household health on CA. The results are as follows. (1) The higher the level of household health is, the less CA there is. (2) Compared with males, the impact of female health status on CA is more obvious. Thus, the relationship between household health and CA matters, not only because it may help to theoretically enhance the understanding of the importance of health in peasant households, but also because it may help to practically provide references for effective policies of CA from the perspective of rural medical services.

## 1. Introduction

**Figure 1:**
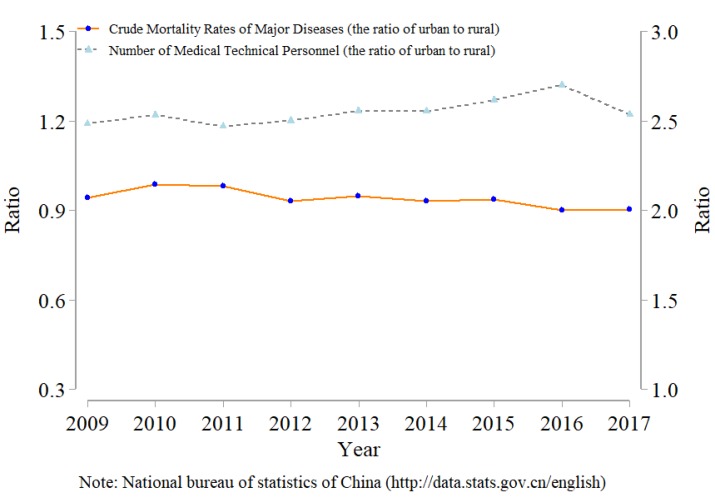
The relationship between health and medical conditions by rural and urban areas.

Scholars have paid attention to poverty and health in developing countries [1,2]. In particular, the problems of the welfare and health of peasant households have drawn substantial attention from scholars. One example of such problems is the decentralized operation of small peasant households, which is the basic form of agricultural production and management in China [3]. According to China’s third agricultural census data [4], at the end of 2016, the share of small peasant households to total peasant households is more than 98%, the share of population engaging in agriculture of small peasant households to total population engaging in agriculture is 90%, and the share of the cropland area of small peasant households to total cropland area is 70%. However, the health status of small peasant households may not be good; specifically, the health status of rural households may be worse than the health status of urban households [5]. As shown in Figure 1, the crude mortality rates of major diseases in rural areas were higher than those in urban areas from 2009 to 2017. Correspondingly, the basic medical conditions in rural areas are not as suitable as those in urban areas [6]. As shown in Figure 1, there were fewer medical technical personnel in rural areas than in urban areas from 2009 to 2017.

Additionally, with the rapid development of the economy and society, CA has become a part of the global agricultural landscape [7,8]. Campbell et al. [9] found that the area of CA has grown to approximately 385–472 million km^2^ worldwide since the 20th century. In recent years, the problem of CA in China has also become serious. In China, big data show that approximately 12% of the small peasant households have abandoned cropland at the end of 2013 [10]. In particular, the problem of CA is more serious in hilly and mountainous areas, where the natural environment and medical conditions are poor. For example, in hilly and mountainous areas, big data show that approximately 15% of China’s small peasant households abandoned cropland at the end of 2013 [8]; Li et al. [11] found that approximately 28% of the cropland in mountainous areas had been abandoned from 2000 to 2010. 

It is important to understand CA [8,12]. First, cropland is abandoned suddenly, which will lead to a series of serious environmental problems [13]. Lasanta et al. [14], Arnáez et al. [15], and Brandolini et al. [13] found that CA increases the risk of soil degradation; Bordoni et al. [16], Persichillo et al. [17], and Schilirò et al. [18] considered CA to be one of the important driving forces behind the shallow landslide phenomenon. Second, Fao et al. [19] pointed out that more than 820 million people in the world did not have enough food in 2018, and 2018 is the third year in which the number of the world’s hungry people has continued to grow. However, food security is directly related to the supply of cropland [20,21,22], and CA reduces the supply of cropland, thereby threatening food security. Thus, discussing the driving factors and their mechanisms of cropland abandonment has gradually become an important focus.

Prior studies have mostly explained the problem of CA from the perspective of migration rather than household health. Operating land requires labor [23]. Labor migration will reduce agricultural labor, which leads to CA. Thus, Gellrich et al. [24], Van Doorn and Bakker [25], Lieskovský et al. [26], Xu et al. [27], Deng et al. [10], and Xu et al. [12] found that migration is a key driver of CA. However, even if the migration rate is low, rural cropland may still be abandoned. Thus, CA is related not only to the quantity of the agricultural labor supply, but also to the quality of the agricultural labor supply. Deng et al. [28] found that the health status of householders may affect land-use decision making. In addition, the health status of household members may affect agricultural productivity [29,30], and its path of influence is multifaceted: (1) Health affects human capital accumulation [31], which may affect household sustainable development; (2) health status may also affect the improvement of agricultural models and the adoption of agricultural technology [32,33], which may directly affect agricultural output. Unfortunately, although some studies suggest that household health may affect CA, the quantitative relationship between household health status and cropland use is not well understood. Thus, it is necessary to explore the quantitative impact of the health status of small peasant households on CA from a health quality perspective.

Moreover, China is the largest developing country in the world [10,34]. “A big country with small peasants” is an accurate definition to current Chinese national conditions. The 19th National Congress of the Communist Party of China proposed a rural revitalization plan that aimed to achieve an effective connection between small peasant households and modernization. Based on the above, this study finds that the poor health status and the widespread CA phenomenon overlap in rural China. However, for small peasant households, health is a prerequisite for access to modernization, and cropland is an important material carrier for access to modernization. Is there a correlation between health status and CA? Thus, based on the big survey data in China exploring the quantitative relationship between health status and CA, the results of this study may contribute to the smooth progress of China’s rural revitalization plan and help provide a reference for developing countries to improve the welfare of small peasant households.

## 2. Theoretical Analysis

For the drivers of household land use, the number of household members is an important determinant. More specially, off-farm employment leads peasant households to abandon cropland [10,12]. However, few studies have explored the quantitative impact of household health on CA from the perspective of the health of household members. Nonetheless, health status plays an important role in land use [28]. Household health may affect land use behavior through labor supply [1,2].

Household health may affect the quantity of labor supply [35,36,37], which directly affects land use behavior. The quantity of labor supply refers to the time and size of labor supply. Jack [35] found that unhealthy family members need healthy family members to look after them, so health status directly affects the quantity of the total labor supply; Fink and Masiye [36] found that improving household health improves the quantity of labor supply in rural areas. More specifically, if the quantity of labor supply is decreased, the household may decrease labor input in cropland management. However, the decrease of agricultural labor is the main driving factor of CA [10,12]. In summary, there exists a theoretical chain of “household health to quantity of labor supply to CA”.

Household health may also affect the quality of labor supply [35,38], which indirectly affects land use behavior. The quality of labor supply refers to the productivity of labor supply. Fogel [38] found that healthy people have higher productivity than unhealthy people. Jack [35] found that health status directly affects the quality of labor supply, for example, diseases may reduce productivity of work. In other words, if the quality of labor supply is decreased, household may decrease or abandon labor input in cropland management. In summary, there exists a theoretical chain of “household health to quality of labor supply to CA”.

Moreover, gender exerts different impacts of health status on labor supply for males and females, which implies that the health status of male members and female members may have different effects on cropland use behavior. Due to female migration lagging behind male migration, rural households have a clear labor division [39,40,41,42]. In rural areas, common divisions of labor mean that males engage in the off-farm sector and females in the farm sector. Thus, from the perspective of gender, cropland use behavior may be more affected by female health status.

As mentioned above, whether household health status affects the quantity or the quality of labor supply will ultimately affect household land use behavior [1,2]. Thus, this study proposes two hypotheses as following:

H_1_: The healthier the household is, the less cropland is abandoned.

H_2_: Compared with males, the impact of female health status on CA is more obvious. 

## 3. Data, Variables, and Method

### 3.1. Data Source

The peasant household is an important start for research on the issues of agriculture and rural areas [31,43,44]; thus, this study employs the household-level data of peasant in rural China. The household-level data employed in this study come from a household survey, which was drawn from the China labor-force dynamics survey (CLDS) in 2014 and conducted by the Center for Social Science Survey at Sun Yat-sen University in Guangzhou, China. The data can be found on the web site: http://css.sysu.edu.cn. The survey collects detailed information about China’s social and economic development, including household health, labor migration, and rural land use. To ensure that the sample is nationally representative, the CLDS covers 29 provinces of mainland China (excluding Tibet and Hainan). A multistage cluster, stratified, probability proportional to size (PPS) sampling method is used. The data employed in this study were collected in 2014, which is the most recent year data have been published by the survey institutions. The 2014 survey covers 29 mainland provinces, 209 counties, 401 villages, and 14,214 households. During the data analysis, this study focuses on the household health and CA in rural region, thus, the households living in city are not included in this study. After cleaning the data, data from 8031 valid household questionnaires of 27 provinces are used for the analysis.

### 3.2. Variables

#### 3.2.1. Dependent Variable

Referring to the studies of Deng et al. [10], Deng et al. [8], and Xu et al. [12], this study defines the dependent variable as the share of CA in total cropland (i.e., Equation (1)). In addition, the variable is the time-point data of peasant households at the end of 2013.

(1) $$CA=\frac{Abandoned\;area\;of\;cropland}{Total\;area\;of\;cropland}\times 100\%$$

where abandoned cropland means the plot which the household did not have any inputs (such as labor, fertilizer, pesticides, etc.) throughout the entirety of 2013; total cropland includes the plot that the household received official registration certificates or rented in, but does not include the plot that the household rented out.

#### 3.2.2. Independent Variable

This study defines the health of peasant households as the share of healthy members in the household (i.e., Equation (2)) and defines female (male) health as the share of healthy female (male) members among female (male) household members (i.e., Equations (3) and (4)). Referring to the studies of Currie and Madrian [45] and Li and Zhu [46], this study selects self-rated health as a measure of member health. In the questionnaire, the interviewee will be asked “What is the health status of the member?” and the interviewee can only choose one of the five answers (i.e., very healthy, healthy, general, unhealthy, and very unhealthy). If the interviewee selects either very healthy, healthy, or general, the member is considered a healthy member.

(2) $$\mathrm{Household}\;\mathrm{health}=\frac{Healthy\;members}{Total\;members}\times 100\%$$

(3) $$\mathrm{Female}\;\mathrm{health}=\frac{Healthy\;female\;members}{Total\;female\;members}\times 100\%$$

(4) $$\mathrm{Male}\;\mathrm{health}=\frac{Healthy\;male\;members}{Total\;male\;members}\times 100\%$$

where members mean population who live together or share economic benefits, which include children and elder.

#### 3.2.3. Control Variable

In order to improve the accuracy of the estimation results, this study also control some other variables. Thus, this study divides the control variables into four categories (e.g., land characteristics, householder characteristics, household characteristics, and location characteristics). More specifically, referring to the study of Cavicchioli et al. [47], Deng et al. [10], Zou et al. [48], Cavicchioli et al. [49], and Xu et al. [12], this study controls land characteristic variables (e.g., Scale, Quality, Registration, and Transfer), householder characteristic variables (e.g., Education and Age), household characteristic variables (e.g., Size, Off-farm employment, Successor), and location characteristic variables (e.g., Distance, Density, Urbanization). The model variables and summary statistics are described in Table 1.

### 3.3. Method

The dependent variable CA is a truncated continuous variable (i.e., the minimum value is 0, the maximum value is 100). In addition, there is a potential endogenous problem, which arises from the mutual causality between household health and CA. Thus, this study employs the instrumental variable Tobit (IV-Tobit) model to explore the relationship between household health and CA. The Tobit (IV-Tobit) model is set as Equation (5):

(5) $${\mathrm{CA}}_{ip}=\beta_{0}+\beta_{1}Household\;health_{ip}+\gamma CV+\eta_{p}+\varepsilon_{ip}$$

where the subscripts of i and p represent household and province, respectively; Cropland abandonment is a truncated and continuous variable, which represents the share of CA in total cropland; Household health is a continuous variable, which represents the share of healthy members in household; CV is the vector of control variables; $\beta_{0}$ is the constant; $\beta_{1}\;$ is the estimated parameters, and if $\beta_{1}$ is significantly nonzero, it will mean that household health significantly affects CA; γ is the vector of estimated parameters for control variables; η is the province dummies; ε is the random error terms. In addition, there may be a mutual causal relationship between household health and CA (i.e., household health may affect CA, and CA may also counteract household health). Thus, referring to the study of Wooldridge [50], this study employs the regression of instrumental variable (IV-Tobit). Meanwhile, referring to the study of Sacerdote [51], this study builds instrumental variable based on peer effects. The instrument IV-Household health for Household health is the average share of health members in households other than the household of interest (*n* − 1) in the same village, as Equation (6):

(6) $$\begin{array}{ll} {\mathrm{IV}-\mathrm{Household}\;\mathrm{health}}_{\mathrm{iv}}=\; & (Household\;{health}_{1}\;+\;Household\;{health}_{2}\;+\;\ldots \;+\\ & Household\;{health}_{n-1})/(n-1) \end{array}$$

## 4. Results

### 4.1. Descriptive Statistics by Relationship

**Figure 2:**
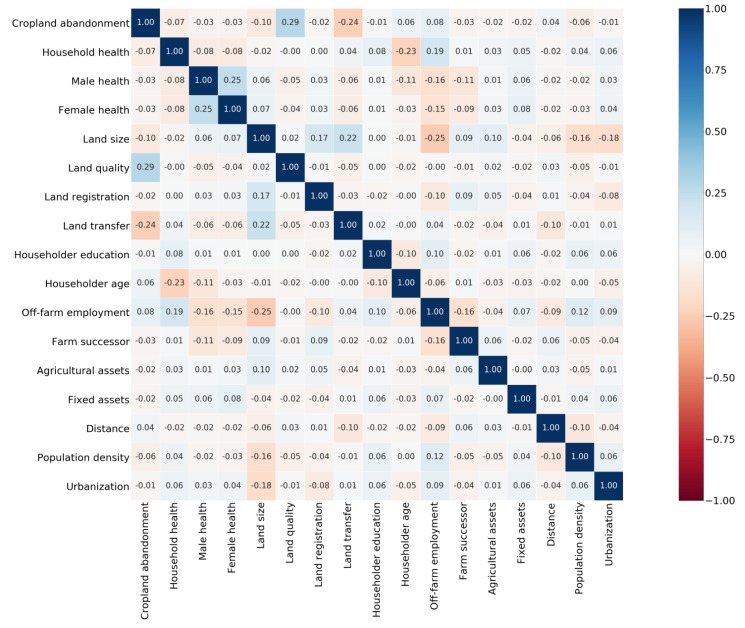
The heatmap for the matrix of Pearson’s correlation coefficients.

Based on the matrix of Pearson’s correlation coefficients for the model variables, we draw the heatmap (Figure 2). The heatmap means that the darker the color of the areas, the bigger the absolute value of the correlation coefficient, and vice versa. As shown in Figure 2, the color of the most areas is light, and the correlation coefficients among the variables are all below 0.3, which indicates that there is no significant multi-collinearity among the variables. Additionally, there is negative correlation between CA and household health (i.e., the value is −0.07). The result also shows that household health may help to reduce CA. However, the results in Figure 2 do not control for other variables when exploring the correlation between the dependent variable and the key variable. Thus, to better test the quantitative relationship, it is necessary to focus on econometric models.

### 4.2. Empirical Results

#### 4.2.1. The Impacts of Household Health on Cropland Abandonment 

Table 2 represents the estimated results of household health affecting CA. This study employs a stepwise regression to explore the quantitative impacts of household health on CA. As shown in Table 2, the dependent variables are the share of CA in all the models. More specifically, for Models (1) to (6), this study gradually adds the key variable, province dummies, land characteristics, householder characteristics, household characteristics, and location characteristics. In all the models, the Wald test values are significant at the 1% or 5% level, which indicates that the null hypothesis (all variables are exogenous) is rejected. More specifically, there is an endogeneity problem, so this study’s use of the IV-Tobit model is appropriate. In addition, since the IV-Tobit model is a nonlinear model, this study also calculates the coefficient of marginal effects (i.e., the column of Model (7)) based on Model (6).

As shown in Table 2, in Models (1) to (6), the coefficients of *household health* are significantly nonzero. More specifically, the coefficients of *household health* are negative and significant at the 1% level in Models (1) to (6), which indicates that the impact of *household health* on *CA* may be robust after solving the problem of missing variables using a stepwise regression. In addition, according to the results of Model (7), every 1% increase in *household health* will result in a 0.15% reduction in *CA*. Therefore, the above estimates provide the empirical evidence for H_1_, namely, with the continuous improvement of household health status, the share of CA gradually decreases.

The stepwise regression can partially ensure the robustness of the estimation results. Hence, to ensure the robustness of the estimation results of Table 2, this study employs the variety of identification strategies shown in Table 3. As shown in Table 3, Model (1) means that the estimation method is replaced by two-stage least squares (linear estimation); Model (2) means that the dependent variable (the share of CA) is replaced by the area of CA; Model (3) means that the dependent variable (the share of CA) is replaced by the binary variable (1 if rural households abandon cropland and 0 otherwise), and the estimated method is replaced by the IV-Probit model. All the models control for province dummies, householder characteristics, household characteristics, and location characteristics. As shown in Table 3, the coefficients of household health have a significant (at the 1% or 5% level) and negative sign, which indicates that there is a significant and negative relationship between household health and CA regardless of changes to the econometric method or the resetting of the explained variables. In other words, the higher the level of household health is, the less CA there is. Thus, the estimates of Table 3 provide further evidence for H_1_.

#### 4.2.2. The Impacts of Household Health on Cropland Abandonment by Gender Composition

As shown in Table 4, the dependent variable is the share of CA in all models. More specifically, for Models (1) to (3), this study gradually adds the key variable, province dummies, land characteristics, householder characteristics, household characteristics, and location characteristics. In all models, the values of the Wald test are significant at the 1% level, which indicates that the null hypothesis (all variables are exogenous) is rejected, and the IV-Tobit model is appropriate.

As shown in Table 4, the estimates of Models (1) to (3) provide evidence for H_2_. Firstly, the coefficients of female health and male health have a negative sign, which suggests that improving female or male health can help reduce CA. Secondly, the absolute value of the coefficient of female health is greater than that of male health, which indicates that female health has a greater impact on CA than male health. Finally, the coefficients of female health in Models (1) to (3) are significant at the level of 1%, and the coefficient of male health is significant only in Model (1), which suggests that the impact of female health status on CA is more robust than that of male health status. In summary, compared with males, the impact of female health status on CA is more obvious. Thus, the estimates of Table 4 provide evidence for H_2_.

## 5. Discussion

This study employs big data via sample surveys from 27 Chinese provinces to explore the quantitative impacts of household health on CA. Compared with prior studies, the marginal contributions of this study are as follows: (1) This study builds the theoretical mechanism of “household health affects CA by labor supply” to theoretically analyze the relationship between household health and CA; (2) this study employs an econometric model to explore the quantitative impacts of household health on CA; and (3) this study also explores the quantitative impacts of the health status of gender composition on CA from the perspective of gender difference. In addition, compared to studies with small-scale sample survey data, this study employs survey data from 8031 households collected in 27 Chinese provinces in 2014, and thus, the results of this study may be more useful in mapping actual problems. Hence, the findings of this study may help provide references for effective cropland management policies from the perspective of rural medical services.

First, the healthier the household is, the less cropland is abandoned. These findings have some similarities and differences with those of prior studies. On the one hand, the findings of this study are similar to the studies of Fogel [38] and Jack [35], which found that healthier the labor force is, the higher productivity is, which also means that peasant households with better health tend to experience less CA. On the other hand, the findings of this study are different from the studies of Currie and Madrian [45] and Goryakin and Suhrcke [52], which found that unhealthy people might not stop working, which also means that health status does not affect the quantity of the labor supply. However, labor supply includes not only the quantity of the supply but also the quality of the supply. Therefore, unhealthy workers may experience low productivity [38,53], which causes peasant households to shrink the scale of their agricultural operations. Therefore, although peasant households with poor health do not stop working, they may reduce the scale of their activities (i.e., they will give priority to high-productivity plots and abandon low-productivity plots) [1,2].

Second, this study finds that female health status is more important for reducing CA than male health status. This finding is different from those of several prior studies. Thomas and Strauss [54], Thomas et al. [55], and García-Gómez et al. [56] believed that males may engage in work that depends on physical status, and thus, the impact of male health status on labor supply or wage is more obvious than that of female health status. However, the findings of this study may be more in line with the current situation in China. In rural China, most men are engaged in off-farm production, and most women are engaged in farm production [57]. Thus, the male health status mainly affects the supply of off-farm labor, and the female health status mainly affects the supply of farm labor. When the level of female health declines, it will directly affect agricultural production, subsequently will increase the risk of CA.

Finally, this study has several deficiencies, which can be addressed in future studies. Specifically, (1) this study measures health status based on self-reporting and finds that peasant households with poor health status are more likely to abandon cropland. Future research can further design scales to measure health status and explore its impact on cropland use. (2) This study focuses on the quantitative relationship between household health and CA. Future research can further explore the implementation mechanisms and approaches behind the quantitative relationship. (3) Household health and CA may be dynamic. Future research can further explore the dynamic relationship between household health and CA using panel data.

## 6. Conclusions and Implications

Under the guidance of the theoretical mechanism of “household health to labor supply to CA”, this study employs survey data from 8031 households collected in 27 Chinese provinces in 2014 to explore the quantitative impacts of household health on CA. This study finds that:

(1) The higher the level of household health is, the less CA there is; specifically, every 1% increase in household health results in 0.15% reduction in CA.

(2) Compared with male, the impact of female health status on CA is more obvious. 

The above results offer several implications. Human health plays an important role in the development of society and the economy. However, in rural areas, the health status of peasant households is a concern due to environmental exposure, poor water quality, and climate change. This study finds that peasant households with better health tend to experience less CA, which also provides evidence of the importance of health. This result implies that the government should pay more attention to rural medical problems. In addition, this study finds that female health is more important in farm production than male health. This result suggests that we should appropriately increase scholarly focus on female health.

## Figures and Tables

**Table 1:** Definition and descriptive statistics for the variables ^a^.

Variables	Definition	Mean	SD
**Dependent variable**			
Cropland abandonment	The share of cropland abandonment in total cropland (%)	7.06	22.74
**Independent variable ^a^**			
Household health	The share of healthy members in household (%)	86.34	23.51
Female health	The share of healthy female members in household (%)	68.75	29.68
Male health	The share of healthy male members in household (%)	73.87	26.95
**Land variables**			
Scale	Per capita of cropland area (mu ^a^ /person)	1.67	1.97
Quality	1 if abandoned cropland is poor; 0 otherwise	0.03	0.16
Registration	1 if cropland is officially registered; 0 otherwise	0.41	0.49
Transfer	1 if household rents out cropland; 0 otherwise	0.71	0.45
**Householder variables**			
Education	1 if householder has a high school education or above; 0 otherwise	0.12	0.32
Age	Age of householder (years)	53.81	13.24
**Household variables**			
Size	Number of total household members	4.61	2.21
Off-farm employment	The share of off-farm labors in total labors (%)	40.01	38.54
Successor	1 if the next generation of householder is engaged in agriculture; 0 otherwise	0.08	0.27
Agricultural assets	Per capita of current market value of all the agricultural assets that a household possesses (10^4^ RMB ^a^/person)	0.08	0.53
Fixed assets	Per capita of current market value of all the fixed assets that a household possesses (10^4^ RMB ^a^/person)	4.32	16.75
**Location variables**			
Distance	Distance from households to the nearest business center (Km)	7.12	9.18
Density	Village population density (number/Km^2^)	140.68	134.30
Urbanization	The share of urban households in total households with same county in sample (%)	11.63	20.69
Plain	1 if village is located in plain; 0 otherwise	0.40	0.49
Hill	1 if village is located in hill; 0 otherwise	0.35	0.48
Mountain	1 if village is located in mountain; 0 otherwise	0.25	0.43

^a^ Note: During the study period, 1 USD was equal to 6.12 RMB; 1 mu ≈ 666.67 m^2^; the gender of some members is a missing value.

**Table 2:** The IV-Tobit estimated results for the impacts of household health on cropland abandonment ^a^.

	Model (1)	Model (2)	Model (3)	Model (4)	Model (5)	Model (6)	Model (7)
Household health	−3.8768 ^***^	−3.7719 ^***^	−3.3716 ^***^	−3.5376 ^***^	−2.9961 ^***^	−2.1950 ^***^	−0.0015 ^***^
	(0.4706)	(0.7612)	(0.6852)	(0.8048)	(0.7681)	(0.8059)	(0.0005)
Land size			5.6424	5.4644	6.5960	4.9781	0.0034
			(7.1495)	(6.9465)	(6.9483)	(6.5851)	(0.0061)
Land quality			212.5419 ^***^	211.9371 ^***^	208.7084 ^***^	205.8077 ^***^	0.1388 ^***^
			(10.6958)	(10.7877)	(10.6689)	(10.3069)	(0.0094)
Land registration			9.8939	10.7815 ^*^	9.1641	9.4428	0.0064
			(6.3402)	(6.4307)	(6.2380)	(6.3351)	(0.0041)
Land transfer			−86.0644 ^***^	−86.3041 ^***^	−98.0307 ^***^	−95.5968 ^***^	−0.0644 ^***^
			(7.6719)	(7.7422)	(7.7928)	(7.6508)	(0.0053)
Householder education				12.8480	5.3303	5.0150	0.0034
				(9.2065)	(8.8146)	(8.8114)	(0.0059)
Householder age				−2.8156 ^**^	−3.5955 ^***^	−3.5315 ^***^	−0.0024 ^***^
				(1.2364)	(1.1916)	(1.1949)	(0.0008)
Householder age2				0.0218 ^*^	0.0308 ^***^	0.0332 ^***^	0.0000 ^***^
				(0.0121)	(0.0111)	(0.0112)	(0.0000)
Household size					6.1363 ^***^	4.5353 ^***^	0.0031 ^***^
					(1.6149)	(1.6397)	(0.0010)
Off-farm employment					0.6996 ^***^	0.6649 ^***^	0.0004 ^***^
					(0.1003)	(0.1018)	(0.0001)
Farm successor					−11.7624	−12.6754	−0.0085
					(11.0098)	(10.8160)	(0.0074)
Ln(Agricultural assets)					−61.3466 ^***^	−70.2677 ^***^	−0.0474 ^***^
					(21.9725)	(22.0746)	(0.0168)
Ln(Fixed assets)					−5.0069	−6.4852 ^*^	−0.0044 ^*^
					(3.5757)	(3.5607)	(0.0025)
Distance						0.1254	0.0001
						(0.2690)	(0.0002)
Population density						−0.1450 ^***^	−0.0001 ^***^
						(0.0239)	(0.0000)
Urbanization						−0.3144 ^**^	−0.0002 ^**^
						(0.1538)	(0.0001)
Hill						12.7579	0.0086
						(8.0849)	(0.0056)
Mountain						17.6559 ^*^	0.0119 ^*^
						(9.2314)	(0.0065)
Province dummies	No	Yes	Yes	Yes	Yes	Yes	Yes
Wald test of exogeneity	48.8958 ^***^	17.4689 ^***^	16.3676 ^***^	14.8112 ^***^	9.7939 ^***^	3.9856 ^**^	-
Observation	8031	8031	8031	8031	8031	8031	8031

^a^ Note: Standard errors in parentheses; ^*^ *p* < 0.1, ^**^ *p* < 0.05, ^***^ *p* < 0.01; in order to display the estimation results more accurately, this study reserve 4 digits after the decimal point (the same below).

**Table 3:** The estimated results of robustness test for the impacts of household health on cropland abandonment ^a^.

	Model (1)	Model (2)	Model (3)
Household health	−0.1554 ^**^	−0.0211 ^**^	−0.0162 ^***^
	(0.0727)	(0.0095)	(0.0057)
Land variables	Yes	Yes	Yes
Householder variables	Yes	Yes	Yes
Household variables	Yes	Yes	Yes
Location variables	Yes	Yes	Yes
Province dummies	Yes	Yes	Yes
Observation	8031	8031	8031

^a^ Note: Standard errors in parentheses; ^*^ *p* < 0.1, ^**^ *p* < 0.05, ^***^ *p* < 0.01.

**Table 4:** The estimated results for the impacts of household health on cropland abandonment by gender composition.

	Model (1)	Model (2)	Model (3)
Female health	−0.0011 ^***^	−0.0015 ^***^	−0.0033 ^**^^*^
	(0.0002)	(0.0003)	(0.0005)
Male health	−0.0013 ^***^	−0.0004	−0.0020
	(0.0002)	(0.0003)	(0.0014)
Land variables	No	No	Yes
Householder variables	No	No	Yes
Household variables	No	No	Yes
Location variables	No	No	Yes
Province dummies	No	Yes	Yes
Wald test of exogeneity	337.8985 ^***^	101.1544 ^***^	41.5459 ^***^
Observation	7581	7581	7581

^a^ Note: The coefficients is marginal effect based on the IV-Tobit estimated result. Standard errors in parentheses; ^*^ *p* < 0.1, ^**^ *p* < 0.05, ^***^ *p* < 0.01.
